# Diversity of common bean rhizobia in blackland of northeastern China and their symbiotic compatibility with two host varieties

**DOI:** 10.3389/fmicb.2023.1195307

**Published:** 2023-07-06

**Authors:** Ziqi Wang, Lili Liu, Dong Hu, En Tao Wang, Chuntao Gu, Hao Wang

**Affiliations:** ^1^College of Life Science, Northeast Agricultural University, Harbin, Heilongjiang, China; ^2^Institute of Agro-Resources and Environment/Hebei Fertilizer Technology Innovation Center, Hebei Academy of Agriculture and Forestry Sciences, Shijiazhuang, Hebei, China; ^3^Departamento de Microbiología, Escuela Nacional de Ciencias Biológicas, Instituto Politécnico Nacional, Ciudad de México, México

**Keywords:** rhizobia, diversity, novel species, common bean, symbiosis diversity of common bean rhizobia

## Abstract

The common bean (*Phaseolus vulgaris* L.) is an important crop in the world that forms root nodules with diverse rhizobia. Aiming to learn the rhizobial communities associated with the common bean in the black soil of Northeast China, 79 rhizobia were isolated from root nodules of two host varieties (Cuican and Jiadouwang) grown in two sites of blackland and were characterized by comparative sequence analyses of 16S rRNA, *recA, atpD, nodC*, and *nifH* genes, and whole genome. As a result, *Rhizobium indigoferae, R. anhuiense*, and *R. croatiense* as minor groups and three dominant novel *Rhizobium* species were identified based on their average nucleotide identity and DNA–DNA hybridization values to the type strains of relative species. This community composition of rhizobia associated with the common bean in the tested black soils was unique. Despite their different species affiliations, all of them were identified into the symbiovar phaseoli according to the phylogenies of symbiotic genes, *nodC* and *nifH*. While the phylogenetic discrepancies found in *nodC, nifH* evidenced that the evolutions of nodulation (*nod*) and nitrogen fixation (*nif* ) genes were partially independent. In addition, only one dominant rhizobial species was shared by the two common bean varieties grown in the two soil samples, implying that both the plant variety and the soil characteristics affected the compatibility between rhizobia and their hosts. These findings further enlarged the spectrum of common bean-nodulating rhizobia and added more information about the interactions among the soil factors, rhizobial species, and host plants in the symbiosis.

## Introduction

As an important leguminous food crop, the seeds of the common bean (*Phaseolus vulgaris* L.) contain rich nutrients, especially protein and starch. Compared with expensive animal-derived food, common beans are widely consumed in many countries as a cheaper source of protein (Los et al., 2018; Shamseldin and Velázquez, 2020). This legume plant is believed to originate and first domesticated in Mexico and the Andes Mountains, and then, it was widely distributed/planted all over the world (De Ron et al., 2019). During the long history of its cultivation, several secondary diversification centers of the common bean have developed, such as Europe (Rodiño et al., 2003, 2006), Africa (Martin and Adams, 1987; Khairallah et al., 1990), Asia (Zhang et al., 2008), and South America (Maciel et al., 2003). The common bean has been cultivated for more than 400 years in China (Zhang et al., 2008), covering various agricultural areas, including the provinces of Heilongjiang and Guizhou (Yeh et al., 1997). Similar to many other legumes, the common bean can fix nitrogen in symbiosis (root nodules) with diverse rhizobia (Shamseldin and Velázquez, 2020). However, the symbiotic nitrogen fixation capacity of the common bean is lower than other major crop legumes (Herridge et al., 2008), but this capacity may vary depending on the compatibility between the symbiosis partners and their adaption to the local geographic environments (Efstathiadou et al., 2021).

Growing in different geographic regions, common bean plants can nodulate with diverse rhizobia, including species in the genera *Rhizobium, Pararhizobium, Sinorhizobium*, and *Bradyrhizobium* in the class of Alphaproteobacteria and in the genera *Paraburkholderia* and *Cupriavidus* in the class Betaproteobacteria; so, it was defined as a promiscuous host for rhizobia (Shamseldin and Velázquez, 2020). It has been estimated that the symbiosis promiscuity could be a result of the interactions among the rhizobia, host plants, and the environmental factors in their habitats (Cao et al., 2014; Zhang et al., 2018, 2020) because both the hosts and the microsymbionts have to adapt the local conditions. In this case, common bean plants grown in different regions have to select the most adapted/compatible species and symbiovars of rhizobia for their habitats. Up to now, eight symbiovars have been found among the common bean-nodulating rhizobia, including phaseoli, tropici, gallicum, mimosa, orientale, giardini, mediterranense, and unnamed (Efstathiadou et al., 2021). Symbiovar phaseoli is the most common one found in common bean rhizobia (Shamseldin and Velázquez, 2020), and it is one of the symbiovars mainly contained by rhizobia nodulating with the common bean in its center of origin. Symbiovar phaseoli has been found in Asia (Huo et al., 2019), Europe (García-Fraile et al., 2010), and Africa (Mhamdi et al., 2002). Accompanying the distribution of the common bean in the world, *Rhizobium etli* in the symbiovar phaseoli also disseminated by attaching to the seeds (Pérez-Ramírez et al., 1998), and then, the symbiosis genes of this symbiovar might have been acquired by local bacterial strains through horizontal gene transfer (HGT) (Rajnovic et al., 2019; Tong et al., 2020). Previously, rhizobia associated with the common bean have been classified in *Bradyrhizobium diazoefficiens*, four unclassified *Bradyrhizobium* spp., *R. leguminosarum, R. etli* complex (with 4 subgroups), *R. phaseoli, R. vallis*, and four unnamed *Rhizobium* genospecies in the northern region of China (Cao et al., 2014), with *Rhizobium* sp. IV (*R. chutanense*, Huo et al., 2019) as the most abundant genospecies. In Shaanxi province (Loess Plateau), common bean formed nodules with four novel *Rhizobium* genospecies, *R. phaseoli, Pararhizobium giardinii, Sinorhizobium fredii, and S. kummerowiae* and two novel genospecies of *Sinorhizobium* and *Bradyrhizobium liaoningense* (Wang et al., 2016). These studies demonstrated that different rhizobial communities have developed in association with the common bean in distinct Chinese regions. Meanwhile, these studies and some others also revealed that at least three symbiovars with quite different *nodC* types existed in these common bean-nodulating rhizobia, in which symbiovar phaseoli was the main one found in various species (genera) (Wang et al., 2011, 2016; Cao et al., 2014; Huo et al., 2019).

Blacklands with “chernozem” soil in the world are known as the most productive regions for agriculture in relation to their high fertility and rich in humus, mainly located in North America, east Europe, and northeastern China. In these three regions, the black soil in China has characteristics distinct from those in the other two regions in relation to the difference in soil formation procedure. The Chinese black soil was developed from steppe meadow and characterized by the absence of CaCO_3_ but is rich in Fe-Mn nuclei and SiO_2_ powder, while CaCO_3_ is rich in the American and European black soils (https://www.sohu.com/a/515314435_100941). Based on these characteristics, the Chinese blackland formed a special ecosystem, which might exist in unique microbial communities adapting to the local conditions. The blackland in China is located in the connecting area between Heilongjiang province, Jilin province, and Inner Mongolia Autonomous Region, where maize, rice, and soybean are the main crops, and the common bean is also widely cultured as a vegetable crop. Previously, several rhizobial strains associated with the common bean in an agricultural-forestry ecosystem in Heilongjiang were classified as *B. japonicum, R. etli*, and *R. leguminosarum*, which harbored *nodC* genes corresponding to those of soybean nodulating *Bradyrhizobium* and symbiovar phaseoli (Wang et al., 2009). However, the rhizobial community associated with the common bean in the black soil in China has not been studied.

Considering the biogeographic patterns and the great diversity of common bean rhizobia uncovered previously, it could be estimated that a unique rhizobial community might exist in the Chinese black soils. To verify this estimation and investigate the compatibility of the native rhizobia with different common bean varieties, rhizobia isolated from two cultivars of the common bean grown in black soils at two sampling sites in Heilongjiang province were characterized in this study. The results evidenced that rhizobia in black soils formed a unique community characterized by the dominance of three unnamed *Rhizobium* genospecies belonging to symbiovar phaseoli.

## Materials and methods

### Soil samples and host plants

Soil samples used in this study were collected from 0 to 30 cm in depth in the Spring of 2021 at two sites without rhizobial inoculation history in Heilongjiang province, including Binxian county (N45°48'10”, E127°02'45”) and Muling county (N40°30'16”, E130°15'23”) (Supplementary Figure S1). After being transported to the laboratory at environmental temperature, a part of regular soil samples was air dried, sieved through 100 mesh sieve, and stored at room temperature for the subsequent physiochemical characterization by the standard methods (Bao, 2000). Total nitrogen and total carbon were estimated by the Northeast Institute of Geography and Agroecology, Chinese Academy of Sciences. Total potassium was assayed with Inductively Coupled Plasma-Mass Spectrometry by the equipment service platform at Northeast Agriculture University (Harbin City). The remaining soils were stored shortly in the shade for rhizobial trapping with host plants. As host plants to trap the rhizobia, seeds of common bean varieties named Cuican and Jiadouwang were purchased from the local market. Cuican is a local variety adapted to black soils with characteristics of high-quality vegetable products, e.g., rich in nutrition and less in fiber. The Jiadouwang variety is widely cultivated in China, with a high yield, thick pods, little fiber, and fresh and tender quality.

### Rhizobial trapping with host plants

To extract rhizobia from the soil, a plant trapping experiment was performed (Vincent, 1970). In brief, seeds of both common bean varieties were surface sterilized in 3.5% (w/v) sodium hypochlorite for 2 min, washed with sterile water seven times, and germinated on agar-water (0.8%) plates in darkness at 28°C (Vincent, 1970). A pot of 1 L volume was full-filled with a mixture of soil and sterile vermiculite (1:3 in volume) that was moistured with a nitrogen-free nutrient solution (Vincent, 1970). Then, a germinated seed was transplanted into each of the pots. For each soil sample, 4 pots were cultured in a greenhouse at 20–25°C under a 12/12 h day/night cycle, and sterile water was added regularly.

### Rhizobial isolation

Plants were harvested after 40 days of growth, and 10 pink nodules were selected randomly from each plant for rhizobial isolation. The root nodules were soaked in 95% (v/v) alcohol for 30 s, disinfected with 3.5% (w/v) sodium hypochlorite for 2 min, and then rinsed with sterile water for seven times. The final rinsed sterile water was inoculated on Yeast-Mannitol-Agar (YMA) (Vincent, 1970) to verify the efficiency of surface sterilization. Each root nodule was crushed with 100 μl sterile water, and then, 30 μl suspension was inoculated on the plate of YMA by streaking. After incubation at 28°C for 3–7 days, a colony from each plate was picked and streaked on a new plate for purification. The rhizobial strains isolated from the Jiadouwang variety in Binxian and Muling soils were designated with BJ and MJ codes, and those from the variety Cuican in Binxian and Muling soils were designated with BC and MC codes. A total of 79 strains of rhizobia were isolated from 80 nodules. All the rhizobial isolates were preserved by stock culture in glycerol (30%, w/v) at −80°C.

### Gene sequencing and phylogenetic analyses

All the rhizobial isolates were cultured in TY broth (tryptone 5.0 g, yeast extract 3.0 g, CaCl_2_, 0.6 g, distilled water 1.0 L, and pH 7.0–7.2) at 28°C for 1–2 days, and the biomass was collected by centrifugation for genomic DNA extraction using DNA extraction kit (Tiangen). Housekeeping genes of 16S rRNA, *recA* (DNA recombination/repair protein) and *atpD* (ATP synthase β-subunit), nitrogen-fixing gene *nifH* (dinitrogenase reductase), and nodulation gene *nodC* (N-acetylglucosaminyltransferase) were amplified from the genomic DNA using corresponding primers (Supplementary Table S1) under specific conditions. Amplicons were verified by electrophoresis in 1% (w/v) agarose gel and sequenced commercially by Sangon Biotech (Shanghai) Co., Ltd. The acquired sequences were searched on Blastn of the National Center for Biotechnology Information (NCBI) server (http://www.ncbi.nlm.nih.gov/blast) to obtain closely related sequences of the reference strains. All the acquired sequences and the reference sequences retrieved from the GenBank/EMBL database (http://www.ebi.ac.uk/Tools/sss/fasta/nucleotide.html) were used for phylogenetic analysis based on the List of Prokaryotic Names with Standing in Nomenclature (LPSN) (http://www.bacterio.net) (Parte, 2018). Sequence similarity alignment was performed using the EzTaxon-e server (https://www.ezbiocloud.net/tools/pairAlign) (Yoon et al., 2017). Sequences were trimmed and concatenated appropriately using DNAMAN 7.0, and phylogenetic trees were constructed using the neighbor-joining (NJ) method in the MEGA 5.0 software package (Saitou and Nei, 1987). Isolates sharing identical *nifH* or *nodC* gene sequences were defined as the corresponding genotypes. The nucleotide sequences of rRNA were deposited in the GenBank database with accession numbers OP869867–OP869945. The nucleotide sequences of housekeeping genes and symbiosis genes were deposited in the China National Microbiology Data Center (NMDC) (https://nmdc.cn/resource/genomics/sequence) with accession numbers NMDCN0001CUS- NMDCN0001D1A for *recA*, NMDCN0001D1Q- NMDCN0001D48 for *atpD*, NMDCN0001CSI- NMDCN0001CUR for *nifH*, and NMDCN0001CQK-NMDCN0001CSH for *nodC*. These accession numbers are presented in Supplementary Table S2.

### Genome sequencing and analysis

Whole-genome sequencing was conducted on the Illumina NovaSeq platform of Shanghai Personalbio Technology Co., Ltd. for representative strains selected based on the grouping results of housekeeping gene phylogeny. The sequencing data of the removed joint sequence were assembled from the beginning, as described by Coil D and BankevichA (Conesa and Götz, 2008; Coil et al., 2015). Digital DNA–DNA hybridization (DDH) was applied with the online tool (Genome-to-Genome Distance Calculator 3.0; http://ggdc.dsmz.de/ggdc.php) using formula 2 (Meier-Kolthoff et al., 2013). Whole-genome average nucleotide identity (ANI) was calculated with the online tool (www.ezbiocloud.net/tools/ani) (Yoon et al., 2017). The genome tree was constructed using the service of PATRIC (patricbrc.org/app/PhylogeneticTree). The whole genome sequences have been deposited in the GenBank database and China National Microbiology Data Center (NMDC), which are listed in parentheses in the corresponding phylogenetic trees.

### Nodulation tests

According to the results of species affiliation and the nodulation gene typing, MC62 (*Rhizobium* sp. I), BC49 (*Rhizobium* sp. II), MC63 (*Rhizobium* sp. III), and MC79 (*R. croatiense*) as representative strains were selected for nodulation test. These strains represent the isolates in the novel common bean symbionts and the isolates belonging to the *nodC* gene type (e) differing from that (d) of the defined common bean rhizobia (*R. etli, R. acidisoli*). The nodulation tests were performed by inoculating the common bean seedlings grown in sterile vermiculite moistured with a nitrogen-free nutrient solution as mentioned in rhizobial trapping. Germinated seeds were inoculated with 1 ml of rhizobial culture (OD_600_ = 2.0) in the TY broth. After 4 weeks of growth in the greenhouse, nodulation was observed, and plants with pink nodules were considered effective nitrogen fixation, otherwise, the nodules were considered ineffective (Vincent, 1970).

### Statistical analysis

Ecoregional rhizobia community diversity was evaluated by the Shannon–Weaver index (*H*) and species richness (*d*) with the formula: H = (*N*log*N*+Σ*n*_i_log*n*_i_)*C*/*N*; d=(*S*-1)/log*N*. *N* stands for the number of total isolates in the ecoregion; *n*_*i*_ stands for the number of isolates in genospecies *I*; *C* stands for a constant of 2.3; and *S* stands for the number of genospecies detected in the ecoregion.

## Results

### Soil characteristics

The physiochemical characteristics of the two soils are presented in Table 1. In brief, soil from Binxian was more acidic (pH 5.86) with less nutrients (TOC, TN, and AP) than that from Muling presented with a pH of 6.1 and greater TOC, TN, and AP but less K.

**Table 1:** Soil characters, rhizobial diversity, and rhizobial distribution against two common bean cultivars in the two sampling regions.

**Soil characteristics**	**Sampling site**			

	**Binxian**		**Muling**	
Value of pH	5.86		6.14	
Total carbon (%)	4.4		5.31	
Total nitrogen (%)	0.32		0.41	
Total potassium (mg/kg)	16,648		12,110	
Available phosphor (mg/kg)	5.338		19.025	
Shannon-Weaver index (*H*)	0.912		0.858	
Species richness (*d*)	1.886		1.257	
**Rhizobial species**	**Cultivar of bean**			
	**Cuican**	**Jiadouwang**	**Cuican**	**Jiadouwang**
*Rhizobium* sp. I (63.29%)	10	16	12	12
*Rhizobium* sp. II (12.66%)	8	2	0	0
*Rhizobium* sp. III (15.19%)	0	0	5	7
*R. indigoferae* (sp. IV, 3.80%)	2	1	0	0
*R. anhuiense* (sp. V, 1.26%)	0	1	0	0
*R. croatiense* (sp. VI, 3.80%)	0	0	2	1
Total: (100%)	20	20	19	20

### Isolation and 16s rRNA gene phylogeny of rhizobia

In this study, 20 isolates were obtained from each of the combinations of soil varieties, Binxian-Cuican (BC), Binxian-Jiadouwang (BJ), and Muling-Jiadouwang (MJ), and 19 isolates were obtained from the combination of soil variety, Muling-Cuican (BC) (Table 1). In the phylogenetic tree of the 16S rRNA gene, all 79 isolates were divided into five genotypes within the genus *Rhizobium* (Supplementary Figure S2). Strains within types A (4 isolates), B (10 isolates), C (5 isolates), and D (45 isolates) were grouped into *R. leguminosarum* complex, together with species *R. anhuiense* CCBAU 23252^T^, *R. indigoferae* CCBAU 71042^T^, and so on, at the similarity ≥99.9%. Type E (15 isolates) showed 100% similarity with *R. croatiense* 13^T^.

### Phylogenetic analysis of *recA* and *atpD* gene sequences

**Figure 1:**
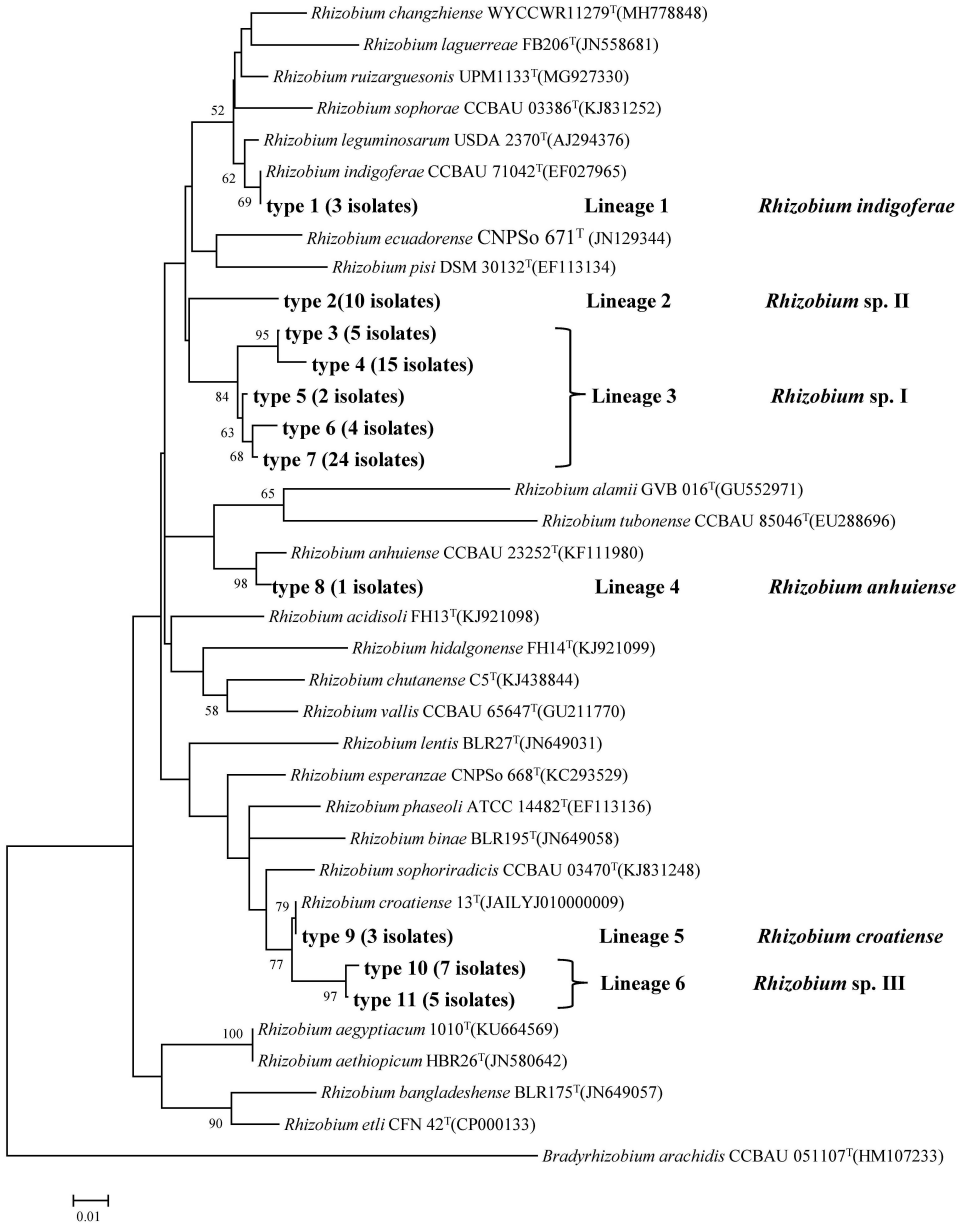
Neighbor-joining tree showing the phylogenetic relationships of genotypes and phylogenetically related reference strains based on the aligned 246bp *recA* gene sequences. Bar, 2% substitutions per site. *Bradyrhizobium* was taken as an outgroup. Higher than 50% bootstrap values are displayed.

In this analysis, *recA* and *atpD* genes were successfully amplified and sequenced for all 79 isolates. Based on the phylogenetic tree of the *recA* gene, 11 genotypes were identified, which could be divided into six lineages or genospecies (Figure 1, Table 2). Type 1 (Lineage 1 with 3 isolates) shared 100% similarity with *R. indigoferae* CCBAU 71042^T^. Type 2 (Lineage 2 with 10 isolates) was a divergent lineage distantly related to *R. ecuadorense* and *R. indigoferae*. Types 3 to 7 (Lineage 3 with 50 isolates) were clustered into a large independent branch showing 97.4–99.5% similarities among them and < 95.5% similarities to the reference strains and the isolates in other *recA* types. Type 8 (Lineage 4 with 1 isolate) grouped with *R. anhuiense* CCBAU 23252^T^. Type 9 (Lineage 5 with 3 isolates) showed 100% similarity with *R. croatiense* 13^T^. Types 10 and 11 (Lineage 6 with 12 isolates) were clustered at 99.53% similarity as an independent branch, and their highest similarity with defined species was 96.98–97.21% with *R. croatiense* 13^T^.

**Table 2:** Genomic typing of the tested strains based on the housekeeping and symbiosis genes.

**Genospecies**	**Genotypes**						Strains^*^
	**rRNA**	***recA***	***atpD***	***recA-atpD***	***nifH***	***nodC***	
*Rhizobium* sp. I	C	3	II	I	a	e	MJ32, MJ34, MJ35^#^, MJ37, MJ40
	D	4	III				BJ11, BJ12, BJ13, BJ14, BJ16, BJ17, BJ18, BC45, BC46, BC47, BC52, BC54, BC56, BC57
		4	II				MJ26
		5					BJ04, BJ08
		6					MJ33, MC65, MC67
		7					BJ01, BJ02, BJ07, BJ09, BJ10, BJ19, BJ20, MJ28, MJ29, MJ36, MJ38^#^, BC53, BC55, BC60, **MC62**, MC64, MC68, MC69, MC70, MC71, MC72, MC74, MC75, MC76
		6			c		MJ22
*Rhizobium* sp. II	B	2	V	V	b	d	BJ15, BC43, BC48, BC51, BC58, BC59
					a	e	BJ05, BC41, **BC49**, BC50
*Rhizobium* sp. III	E	10	VII	VI	a	e	MJ21, MJ23, MJ24, MJ27, MJ30, MC73, MC78
		11					MJ25^§^, MC61, **MC63**, MC66
					c		MJ31
*R. indigoferae* (sp. IV)	A	1	VI	III	a	e	BJ03, BC42, BC44
*R. anhuiense* (sp. V)	A	8	IV	IV	a	e	BJ06
*R. croatiense* (sp. VI)	E	9	I	II	a	e	MJ39, MC77, **MC79**

^*^In strain codes, the first letter represents the sampling site of soil: B, Binxian; M, Muling; while the second letter represents the cultivar of common bean used as tramp host; C, Cuican; J, Jiadouwang. Strains in bold face were used for inoculation test. ^#^ Strains failed for *nifH* and *nodC* amplification. ^§^ Strain failed for *nodC* amplification.

**Figure 2:**
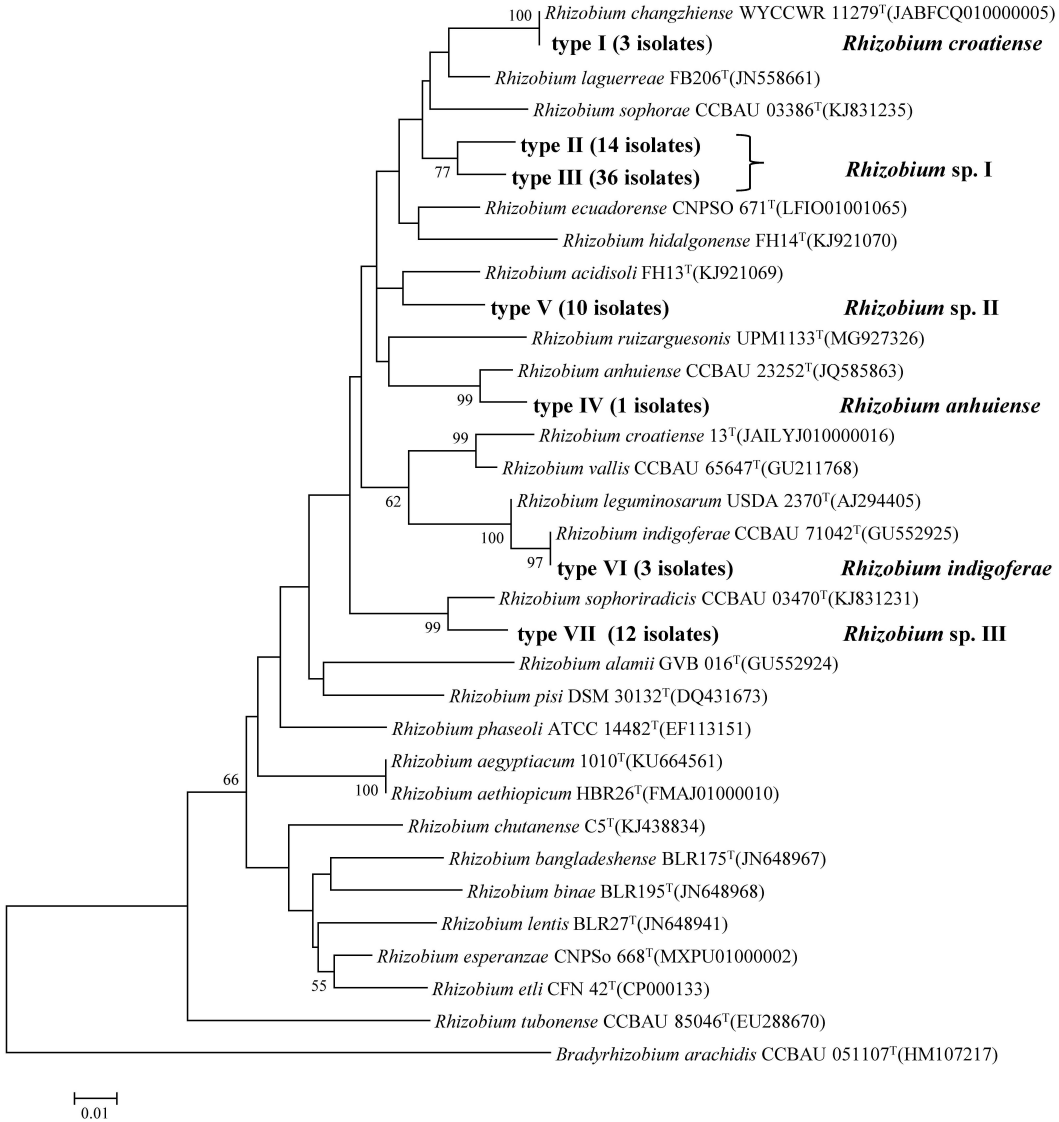
Neighbor-joining tree showing the phylogenetic relationships of genotypes and phylogenetically related reference strains based on the aligned 334bp *atpD* gene sequences. Bar, 1% substitutions per site. *Bradyrhizobium* was taken as an outgroup. Higher than 50% bootstrap values are displayed.

In the phylogenetic tree of the *atpD* gene, seven genotypes were identified that were also clustered into six lineages or genospecies (Figure 2, Table 2). Most of the isolates showed grouping results (topology) similar to that in *recA* phylogeny, except for the three isolates in Type I (Lineage 5 or *recA* type 9). These three isolates were clustered with *R. changzhiense* WYCCWR 11279^T^ at 100% similarity, which was inconsistent with the phylogeny of the *recA* gene.

In the phylogenetic tree based on the concatenated sequences of these two genes (Supplementary Figure S3), the isolates were also grouped into six genospecies with tree topology similar to that of the *atpD* gene alone. So, they were identified as six genospecies corresponding to *Rhizobium* sp. I covering 50 strains; *Rhizobium* sp. II covering 10 strains; *Rhizobium* sp. III covering 12 strains; *Rhizobium* sp. IV with 3 strains related to *R. indigoferae*; *Rhizobium* sp. V covering only strain, BJ06 related to *R. anhuiense*; *Rhizobium* sp. VI with 3 strains related to *R. croatiense* in *recA* and *R. changzhiense* in *atpD*.

### Genome sequencing and phylogenetic analysis

**Figure 3:**
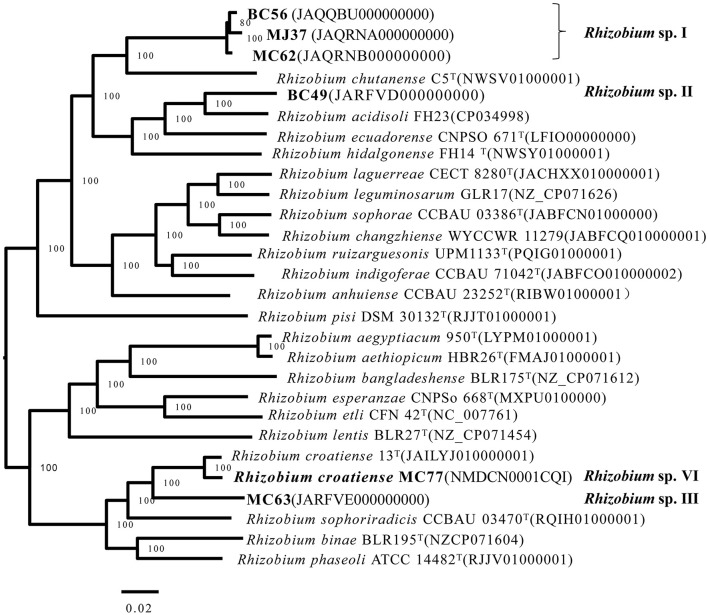
Neighbor-joining tree based on ANI among the genome sequences of tested strains and the type strains of closely related species. Bar, 2% nucleotide substitutions behalf per site. The bootstrap values of >50% are shown in the internodes.

Whole-genome sequencing was performed for representative strains of genospecies, including *Rhizobium* sp. I BC56, MJ37, and MC62; *Rhizobium* sp. II BC49; *Rhizobium* sp. III MC63; and *Rhizobium* sp. VI MC77 (Figure 3). The specific calculated values of ANI and DDH between strains and type strains are shown in Supplementary Table S3. *Rhizobium* sp. VI MC77 could be identified as *R. croatiense* that presented 85% relatedness in DDH and 98.96% of ANI with *R. croatiense* MC 77, supporting the phylogenetic relation in Figure 3. The whole genomes of BC56/MJ37/MC62, BC49, and MC63 formed three lineages distinct from those of type strains for defined species, and they also presented DDH and ANI values lower than the threshold for species, 70 and 95%, respectively, with the reference strains for defined species. Strains BC56, MJ37, and MC62 shared 91.9–93% relatedness in DDH and 99.0–99.2% in ANI, indicating that these three strains belong to the same species. In brief, three potential novel species exist in the rhizobia associated with the common bean in the black soil of China.

### Phylogenetic analysis of *nodC-nifH* genes and nodulation tests

**Figure 4:**
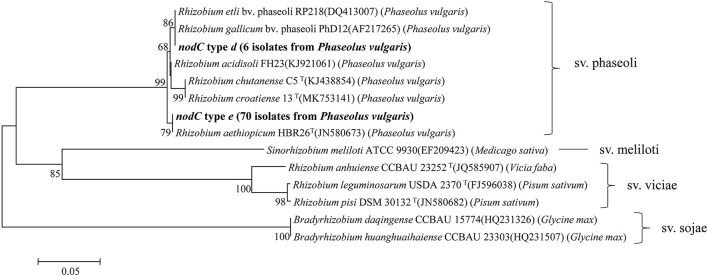
Neighbor-joining tree showing the phylogenetic relationships of genotypes and phylogenetically rhizobia strains from nodules of different legumes based on the aligned 431bp *nodC* gene sequences. Bar, 5% substitutions per site. Higher than 50% bootstrap values are displayed.

Symbiosis genes were used to estimate the symbiovar of isolates in this study. In total, the *nifH* gene and *nodC* gene were amplified from 77 and 76 strains (Table 2), respectively, which were also sequenced successfully. Based on the phylogenetic analyses, three *nifH* genotypes (Supplementary Figure S4) and two *nodC* genotypes (Figure 4) (Table 2 for details) were differentiated among the strains. The *nifH* type *a* was dominant (67 isolates) that was detected in all six genospecies and showed 100% similarity with the *nifH* of *R. acidisoli* FH23. Type *b* was only found in six strains of *Rhizobium* sp. II and showed 100% similarity with *nifH* of *R. etli* sv. phaseoli RP212. Type *c* was defined for one strain of *Rhizobium* sp. I and one strain of *Rhizobium* sp. III and was 100% similar with *nifH* of *R. chutanense* C5^T^ and *R. aethiopicum* HBR26^T^. In fact, this type of highest similarity phenomenon also appeared in the analysis of the *nodC* gene. Type *d* of the *nodC* gene covered six isolates of *Rhizobium* sp. II (with *nifH* type *b*) and showed 100% similarity with that of sv. phaseoli strains in *R. etli* and *R. gallicum*. Type *e* was dominant covering 70 isolates distributed in all the six genospecies and showed 100% similarity with that of *R. aethiopicum* HBR26^T^. In nodulation tests, all four representative strains of *Rhizobium* sp. I MC62, *Rhizobium* sp. II BC49, *Rhizobium* sp. III MC63, and *R. croatiense* MC79 belonging to the *nodC* type e formed red (effective) spherical nodules.

### Distribution of rhizobial species in two sites and their compatibility with host varieties

The diversity indicator of common bean rhizobia was greater in Binxian (*H* = 0.912; *d* = 1.886) than those in Muling (*H* = 0.858; *d* = 1.257) (Table 1). Four species from Binxian and three species from Muling were isolated, and only *Rhizobium* sp. I appeared in both regions as the dominant symbionts, accounting for 63.3% of the total number of isolates and both varieties of the common bean and both regions (Table 1). At the species level, *Rhizobium* sp. II, *R. indigoferae* (sp. IV), and *R. anhuiense* (sp. V) were found only in Binxian soil, while *Rhizobium* sp. III and *R. croatiense* (sp. VI) were only trapped in Muling soil. At the genotype level in *Rhizobium* sp. I, *recA* type 3 and type 6 were only isolated from Muling soil, while type 4 was mainly isolated from Binxian soil. Thus, the rhizobial communities were obviously different in the two sampling sites.

In addition, the difference in compatibility between the rhizobial species and common bean varieties was found in Binxian soil, but it is not clear in Muling soil (Table 1). It seems that *Rhizobium* sp. II was more compatible with the variety Cuican, and *Rhizobium* sp. I was more compatible with the variety Jiadouwang. Furthermore, *R. anhuiense* (sp. V) was only trapped by the variety Jiadouwang at a low frequency (1 isolate).

## Discussion

This study was performed in order to learn the diversity of common bean rhizobia in the black soils in China and their symbiosis compatibility with the host varieties. Based on phylogenetic analyses of housekeeping genes (16S rRNA, *atpD*, and *recA*) and the symbiosis genes (*nodC* and *nifH*), 79 rhizobial strains isolated from two soil samples in association with a local cultivar and a widely cultured cultivar were classified into six genospecies, including three defined species and three novel genospecies (Table 1; Figures 1–3). This classification was verified by the genome analysis for the three novel species. Among these genomic species, the three unclassified genospecies *Rhizobium* sp. I (63.29%), *Rhizobium* sp. II (12.66%), and *Rhizobium* sp. III (15.19%) were dominant, while *R. indigoferae, R. anhuiense*, and *R. croatiense* had relative abundances of 3.80, 1.26, and 3.80%, respectively. This community composition was unique for the studied black soils in China compared with those reported in other studies. For example, common bean nodulated (1) with *R. etli, R. leguminosarum*, and *B. japonicum* in a forest region in Heilongjiang, which also has acid soil with pH 5.3–5.5 (Wang et al. 2009); (2) with *R. leguminosarum* bv. phaseoli in neutral and alkaline soils and *Bradyrhizobium* sp. in acidic soils (pH < 6.0) of the subtropical region of China (Han et al., 2005); (3) with *Sinorhizobium meliloti* sv. mediterranense and *Sinorhizobium americanum* in alkaline soils (Mnasri et al., 2007; Verástegui-Valdés et al., 2014), and *Rhizobium acidisoli/Rhizobium hidalgoense* in acidic soils in other countries (Verástegui-Valdés et al., 2014) and so on. This unique rhizobial community also differed from those mentioned in the introduction (Cao et al., 2014; Wang et al., 2016; Huo et al., 2019). Thus, it could be estimated that common bean plants may have selected some indigenous bacteria as their microsymbionts in the studied black soils. In this study, three dominant unnamed genospecies and *R. indigoferae* were described as microsymbionts of the common bean for the first time, since their nodulation ability on the common bean was verified by the nodulation test with the representative strains and/or by the *nodC* gene phylogeny. Previously, the phylogeny of *nodC* and *nodA* has been used to determine the host spectra and symbiovar of rhizobia, and all the strains belonging to symbiovar phaseoli could nodulate common bean (Efstathiadou et al., 2021; Gunnabo et al., 2021). However, the findings in this study further enlarged the spectrum of common bean-nodulating rhizobia and added novel information about the interactions among the soil factors, rhizobial species, and host plants.

In this study, 16S rRNA, *recA*, and *atpD* genes, as well as the whole genome sequences, were used to define the species, since they have been suggested as valuable biomarkers for differentiating rhizobial species (Zhang et al., 2018, 2020; Tong et al., 2020). In most cases, the grouping results in the phylogenetic trees constructed with the individual genes were similar and were well supported by the whole genome phylogeny (Figures 1–3). However, some discrepancies were observed between the phylogenetic trees of *recA* and *atpD* genes. First, the *recA* gene was more sensitive to estimating the genomic diversity than the *atpD* gene, since more genotypes were identified by *recA*, which gave higher taxonomic resolution. For example, *R. indigoferae* and *R. anhuiense* shared the same sequences in the 16S rRNA gene but they could be distinguished by *recA* sequence analysis. Thus, the results in this study and the previous study demonstrated that < 97% of *recA* gene sequences could be used to primitively distinguish species (Wang et al., 2019). Second, three strains MJ39, MC77, and MC79 in genospecies VI shared identical *recA* gene sequences with the type strain of *R. croatiense*, but their *atpD* genes were identical with that of *R. changzhiense* type strain. Based on the whole-genome sequencing analysis, these three strains were identified as *R. croatiense*. Thus, it could be estimated that the *atpD* gene of these three strains was acquired from *R. changzhiense* by lateral transfer. This case remained us that it is better to define rhizobial species based on the analysis of multilocus genes.

As mentioned above, the symbiosis promiscuous property of the common bean could be seen from both the species level and the symbiovar level. Previously, eight symbiovars have been reported across all the common bean-nodulating rhizobia in different species and genera (Efstathiadou et al., 2021), while the horizontal transfer of symbiosis genes across species and genera has been evidenced or estimated (Tong et al., 2020). In this study, all of them were identified into one symbiovar, e.g., sv. phaseoli (Figures 4 and Supplementary Figure S4), which might demonstrate that (1) horizontal transfer of symbiosis genes might have happened in the local rhizobial communities; and (2) the symbiosis genes of sv. phaseoli might have been introduced from the center of origin of the common bean, along with the plant seeds (Pérez-Ramírez et al., 1998; Tong et al., 2020). Moreover, this may support the hypothesis that the common bean was introduced into China directly from America, and China may be the secondary diversity center of the common bean (Zhang et al., 2008).

In this study, *nifH* and *nodC* genes were amplified from most of the strains, but three of them failed for amplification of both genes or *nodC*, which might be due to primer mismatching or poor DNA quality (De Castro et al., 2018). It is also possible that there are no symbiotic endophytes in nodules since no symbiotic rhizobial strains have been found in soils (Soenens and Imperial, 2018) and root nodules (Moyano et al., 2017). In this experiment, the genotypes of the *nifH* gene and *nodC* gene were inconsistent, and the number of *nifH* genotypes was more than that of *nodC* genotypes. The phenomenon that the *nifH* gene was not synchronized with the *nodC* gene and evolves independently also conforms to the hypothesis that the origin of nitrogen fixation is earlier than nodulation, and the distribution of the *nifH* gene is more extensive (Dos Santos et al., 2012).

Another interesting finding in this study is that the compatibility between common bean cultivars and the rhizobial species might be a feature related to the soil conditions. It seems both the varieties used in this experiment are capable to nodulate all six rhizobial species, but they showed different affiliations with *Rhizobium* sp. I and *Rhizobium* sp. II strains in soils from Binxian but not in the soil of Muling (Table 1). In Binxian soil, the local cv. Cuican *Rhizobium* sp. I did not show preference between the two rhizobial genospecies (10:8), but the widely cultured cv. Jiadouwang preferred sp. I more than sp. II (16:2). Therefore, the symbiosis compatibility between legume and rhizobia might be determined by the genetic backgrounds of the host plant and rhizobial species (Wu et al., 2020; Jiao et al., 2022) but also regulated by the soil conditions, including both the physiochemical features and the local microbiome in soil (Zhang et al., 2018, 2020). In this case, the symbiosis compatibility might also be related to the biogeography or adaptation (Zhang et al., 2018, 2020) and nodulation competition (Jia et al., 2013) of rhizobia.

Previously, soil pH has been reported as a key factor to determine the geographic distribution or nodule occupation of rhizobial species, such as soybean, which mainly nodulate with *Bradyrhizobium* species in acidic soils and with *Sinorhizobium* in alkaline–saline soils (Zhang et al., 2011). A similar situation was also observed in common bean rhizobia (Han et al., 2005; Mnasri et al., 2007; Wang et al., 2009, 2016; Cao et al., 2014; Verástegui-Valdés et al., 2014; Huo et al., 2019). In this study, a clear difference in the geographic distribution of rhizobia was observed on *Rhizobium* sp. II and *Rhizobium* sp. III: the former was only in Binxian soil as the second abundant group; while the latter was only in Muling soil as the second abundant group. It was possible that *Rhizobium* sp. II was more adapted to acidic soil with lower P, while *Rhizobium* sp. III was contrast (Table 1). The soils' pH values in the studies of Wang et al. (2009) and Verástegui-Valdés et al. (2014) were similar to that of the soil from Binxian in this study (around pH 5.5), but the rhizobial communities were quite different from each other; therefore, it can be estimated that soil pH was not the only determinant for rhizobial biogeography (Zhang et al., 2018). The adaptation of rhizobial species to the whole environment and their interaction with other native microbes might also play an important role in the biogeography and compatibility with the host legumes.

## Conclusion

In conclusion, a unique rhizobial community associated with the common bean exists in the studied black soils, which was dominated by three unnamed *Rhizobium* genospecies and three minor species corresponding to *R. indigoferae, R. anhuiense*, and *R. croatiense*. All of them belonged to the symbiovar phaseoli, evidencing that they have coevolved with the host plant under the double selection from soil conditions (for species) and host specificity (for symbiosis genes). In addition, symbiosis compatibility between common bean cultivars and *Rhizobium* species might be altered by soil characteristics. These findings further enlarged the spectrum of common bean-nodulating rhizobia and added novel information about the interactions among the soil factors, rhizobial species, and host plants in the symbiosis.

## Data availability statement

The datasets presented in this study can be found in online repositories. The names of the repository/repositories and accession number(s) can be found in the article/Supplementary material.

## Author contributions

HW was responsible for the design of the experimental scheme. ZW was responsible for the isolation of strains, gene amplification, and paper writing. LL was responsible for the sequencing of the genome. DH was responsible for the potted cultivation of samples. EW was responsible for the modification of the paper. CG was responsible for the analysis of data. All authors contributed to the article and approved the submitted version.
