# Supplementary material for: Diversity of common bean rhizobia in blackland of northeastern China and their symbiotic compatibility with two host varieties
Source: Front Microbiol. 2023 Jul 6;14:1195307. doi: 10.3389/fmicb.2023.1195307 (PMC10362387; doi:10.3389/fmicb.2023.1195307)
Supplement: Supplementary file 1 [file Data_Sheet_1.docx]

**Supplementary Table S1**. Primer sequences and amplification conditions in this study

| Gene | Primer | Sequence **(5' - 3')** | Cycles |
| --- | --- | --- | --- |
| 16S | P1,  P6(Moreno et al., 2002) | AGA GTT TGA TCC TGG CTC AG  GGT TAC CTT GTT ACG ACT T | 5 min 95 °C 30 x (45 s 95 °C, 45 s 58 °C, 75 s 72 °C), 6 min 72 °C |
| *atpD* | *atpD* F,  *atpD* R(Gaunt et al., 2001) | SCT GGG SCG YAT CMT GAA CGT  GCC GAC ACT TCC GAA CCN GCC TG | 5 min 95 °C 30 x (30 s 95 °C, 30 s 58 °C, 30 s 72 °C), 6 min 72 °C |
| recA | *recA* 63,  *recA* 555(Gaunt et al., 2001) | ATC GAG CGG TCG TTC GGC AAG GG  CGR ATC TGG TTG ATG AAG ATC ACC AT | 5 min 95 °C 30 x (45 s 95 °C, 1 min 60 °C, 90 s 74 °C), 6 min 74 °C |
| *nodC* | *nodC* for540,  *nodC* for1160(Laguerre et al., 2001) | TGA TYG AYA TGG ART AYT GGC T  CGY GAC ARC CAR TCG CTR TTG | 3 min 95 °C 30 x (1 min 94 °C, 1 min 58 °C, 1 min 72 °C), 6 min 72 °C |
| *nifH* | *nifH* F 63,  *nifH* I 555(Laguerre et al., 2001) | TAC GGN AAR GGS GGN ATC GGC  AGC ATG TCY TCS AGY TCN TCC A | 3 min 95 °C 30 x (30 s 94 °C, 30 s 58 °C, 1 min 72 °C), 6 min 72 °C |

**Supplementary Table S2.** Sequence accession numbers of different genes in this study.

| Strain | rRNA | *recA* | *atpD* | *nifH* | *nodC* |
| --- | --- | --- | --- | --- | --- |
| BJ01 | OP869867 | NMDCN0001CUS | NMDCN0001D1Q | NMDCN0001CSI | NMDCN0001CQK |
| BJ02 | OP869868 | NMDCN0001CUT | NMDCN0001D1R | NMDCN0001CSJ | NMDCN0001CQL |
| BJ03 | OP869869 | NMDCN0001CUU | NMDCN0001D1S | NMDCN0001CSK | NMDCN0001CQM |
| BJ04 | OP869870 | NMDCN0001CUV | NMDCN0001D1T | NMDCN0001CSL | NMDCN0001CQN |
| BJ05 | OP869871 | NMDCN0001CV0 | NMDCN0001D1U | NMDCN0001CSM | NMDCN0001CQO |
| BJ06 | OP869872 | NMDCN0001CV1 | NMDCN0001D1V | NMDCN0001CSN | NMDCN0001CQP |
| BJ07 | OP869873 | NMDCN0001CV2 | NMDCN0001D20 | NMDCN0001CSO | NMDCN0001CQQ |
| BJ08 | OP869874 | NMDCN0001CV3 | NMDCN0001D21 | NMDCN0001CSP | NMDCN0001DPA |
| BJ09 | OP869875 | NMDCN0001CV4 | NMDCN0001D22 | NMDCN0001CSQ | NMDCN0001CQR |
| BJ10 | OP869876 | NMDCN0001CV5 | NMDCN0001D23 | NMDCN0001CSR | NMDCN0001CQS |
| BJ11 | OP869877 | NMDCN0001CV6 | NMDCN0001D24 | NMDCN0001CSS | NMDCN0001CQT |
| BJ12 | OP869878 | NMDCN0001CV7 | NMDCN0001D25 | NMDCN0001CST | NMDCN0001CQU |
| BJ13 | OP869879 | NMDCN0001CV8 | NMDCN0001D26 | NMDCN0001CSU | NMDCN0001CQV |
| BJ14 | OP869880 | NMDCN0001CV9 | NMDCN0001D27 | NMDCN0001CSV | NMDCN0001CR0 |
| BJ15 | OP869881 | NMDCN0001CVA | NMDCN0001D28 | NMDCN0001CT0 | NMDCN0001CR1 |
| BJ16 | OP869882 | NMDCN0001CVB | NMDCN0001D29 | NMDCN0001CT1 | NMDCN0001CR2 |
| BJ17 | OP869883 | NMDCN0001CVC | NMDCN0001D2A | NMDCN0001CT2 | NMDCN0001CR3 |
| BJ18 | OP869884 | NMDCN0001CVD | NMDCN0001D2B | NMDCN0001CT3 | NMDCN0001CR4 |
| BJ19 | OP869885 | NMDCN0001CVE | NMDCN0001D2C | NMDCN0001CT4 | NMDCN0001CR5 |
| BJ20 | OP869886 | NMDCN0001CVF | NMDCN0001D2D | NMDCN0001CT5 | NMDCN0001CR6 |
| MJ21 | OP869887 | NMDCN0001CVG | NMDCN0001D2E | NMDCN0001CT6 | NMDCN0001DPB |
| MJ22 | OP869888 | NMDCN0001CVH | NMDCN0001D2F | NMDCN0001CT7 | NMDCN0001CR7 |
| MJ23 | OP869889 | NMDCN0001CVI | NMDCN0001D2G | NMDCN0001CT8 | NMDCN0001CR8 |
| MJ24 | OP869890 | NMDCN0001CVJ | NMDCN0001D2H | NMDCN0001CT9 | NMDCN0001CR9 |
| MJ25 | OP869891 | NMDCN0001CVK | NMDCN0001D2I | NMDCN0001CTA | - |
| MJ26 | OP869892 | NMDCN0001CVL | NMDCN0001D2J | NMDCN0001CTB | NMDCN0001CRA |
| MJ27 | OP869893 | NMDCN0001CVM | NMDCN0001D2K | NMDCN0001CTC | NMDCN0001CRB |
| MJ28 | OP869894 | NMDCN0001CVN | NMDCN0001D2L | NMDCN0001CTD | NMDCN0001CRC |
| MJ29 | OP869895 | NMDCN0001CVO | NMDCN0001D2M | NMDCN0001DP7 | NMDCN0001DPC |
| MJ30 | OP869896 | NMDCN0001CVP | NMDCN0001D2N | NMDCN0001CTE | NMDCN0001CRD |
| MJ31 | OP869897 | NMDCN0001CVQ | NMDCN0001D2O | NMDCN0001CTF | NMDCN0001DPD |
| MJ32 | OP869898 | NMDCN0001CVR | NMDCN0001D2P | NMDCN0001CTG | NMDCN0001DPE |
| MJ33 | OP869899 | NMDCN0001CVS | NMDCN0001D2Q | NMDCN0001CTH | NMDCN0001CRE |
| MJ34 | OP869900 | NMDCN0001CVT | NMDCN0001D2R | NMDCN0001CTI | NMDCN0001DPF |
| MJ35 | OP869901 | NMDCN0001CVU | NMDCN0001D2S | - | - |
| MJ36 | OP869902 | NMDCN0001CVV | NMDCN0001D2T | NMDCN0001CTJ | NMDCN0001CRF |
| MJ37 | OP869903 | NMDCN0001D00 | NMDCN0001D2U | NMDCN0001CTK | NMDCN0001CRG |
| MJ38 | OP869904 | NMDCN0001D01 | NMDCN0001D2V | - | - |
| MJ39 | OP869905 | NMDCN0001D02 | NMDCN0001D30 | NMDCN0001CTL | NMDCN0001CRH |
| MJ40 | OP869906 | NMDCN0001D03 | NMDCN0001D31 | NMDCN0001CTM | NMDCN0001CRI |
| BC41 | OP869907 | NMDCN0001D04 | NMDCN0001D32 | NMDCN0001CTN | NMDCN0001CRJ |
| BC42 | OP869908 | NMDCN0001D05 | NMDCN0001D33 | NMDCN0001CTO | NMDCN0001CRK |
| BC43 | OP869909 | NMDCN0001D06 | NMDCN0001D34 | NMDCN0001CTP | NMDCN0001CRL |
| BC44 | OP869910 | NMDCN0001D07 | NMDCN0001D35 | NMDCN0001CTQ | NMDCN0001CRM |
| BC45 | OP869911 | NMDCN0001D08 | NMDCN0001D36 | NMDCN0001CTR | NMDCN0001CRN |
| BC46 | OP869912 | NMDCN0001D09 | NMDCN0001D37 | NMDCN0001CTS | NMDCN0001CRO |
| BC47 | OP869913 | NMDCN0001D0A | NMDCN0001D38 | NMDCN0001CTT | NMDCN0001CRP |
| BC48 | OP869914 | NMDCN0001D0B | NMDCN0001D39 | NMDCN0001CTU | NMDCN0001CRQ |
| BC49 | OP869915 | NMDCN0001D0C | NMDCN0001D3A | NMDCN0001CTV | NMDCN0001CRR |
| BC50 | OP869916 | NMDCN0001D0D | NMDCN0001D3B | NMDCN0001CU0 | NMDCN0001CRS |
| BC51 | OP869917 | NMDCN0001D0E | NMDCN0001D3C | NMDCN0001CU1 | NMDCN0001CRT |
| BC52 | OP869918 | NMDCN0001D0F | NMDCN0001D3D | NMDCN0001CU2 | NMDCN0001CRU |
| BC53 | OP869919 | NMDCN0001D0G | NMDCN0001D3E | NMDCN0001CU3 | NMDCN0001CRV |
| BC54 | OP869920 | NMDCN0001D0H | NMDCN0001D3F | NMDCN0001CU4 | NMDCN0001CS0 |
| BC55 | OP869921 | NMDCN0001D0I | NMDCN0001D3G | NMDCN0001CU5 | NMDCN0001CS1 |
| BC56 | OP869922 | NMDCN0001D0J | NMDCN0001D3H | NMDCN0001CU6 | NMDCN0001CS2 |
| BC57 | OP869923 | NMDCN0001D0K | NMDCN0001D3I | NMDCN0001CU7 | NMDCN0001CS3 |
| BC58 | OP869924 | NMDCN0001D0L | NMDCN0001D3J | NMDCN0001CU8 | NMDCN0001CS4 |
| BC59 | OP869925 | NMDCN0001D0M | NMDCN0001D3K | NMDCN0001CU9 | NMDCN0001DPG |
| BC60 | OP869926 | NMDCN0001D0N | NMDCN0001D3L | NMDCN0001CUA | NMDCN0001CS5 |
| MC61 | OP869927 | NMDCN0001D0O | NMDCN0001D3M | NMDCN0001CUB | NMDCN0001DPH |
| MC62 | OP869928 | NMDCN0001D0P | NMDCN0001D3N | NMDCN0001CUC | NMDCN0001DPI |
| MC63 | OP869929 | NMDCN0001D0Q | NMDCN0001D3O | NMDCN0001CUD | NMDCN0001CS6 |
| MC64 | OP869930 | NMDCN0001D0R | NMDCN0001D3P | NMDCN0001DP8 | NMDCN0001CS7 |
| MC65 | OP869931 | NMDCN0001D0S | NMDCN0001D3Q | NMDCN0001CUE | NMDCN0001CS8 |
| MC66 | OP869932 | NMDCN0001D0T | NMDCN0001D3R | NMDCN0001CUF | NMDCN0001CS9 |
| MC67 | OP869933 | NMDCN0001D0U | NMDCN0001D3S | NMDCN0001CUG | NMDCN0001CSA |
| MC68 | OP869934 | NMDCN0001D0V | NMDCN0001D3T | NMDCN0001CUH | NMDCN0001CSB |
| MC69 | OP869935 | NMDCN0001D10 | NMDCN0001D3U | NMDCN0001CUI | NMDCN0001CSC |
| MC70 | OP869936 | NMDCN0001D11 | NMDCN0001D3V | NMDCN0001CUJ | NMDCN0001DPJ |
| MC71 | OP869937 | NMDCN0001D12 | NMDCN0001D40 | NMDCN0001CUK | NMDCN0001DPK |
| MC72 | OP869938 | NMDCN0001D13 | NMDCN0001D41 | NMDCN0001CUL | NMDCN0001CSD |
| MC73 | OP869939 | NMDCN0001D14 | NMDCN0001D42 | NMDCN0001CUM | NMDCN0001CSE |
| MC74 | OP869940 | NMDCN0001D15 | NMDCN0001D43 | NMDCN0001CUN | NMDCN0001DPL |
| MC75 | OP869941 | NMDCN0001D16 | NMDCN0001D44 | NMDCN0001DP9 | NMDCN0001DPM |
| MC76 | OP869942 | NMDCN0001D17 | NMDCN0001D45 | NMDCN0001CUO | NMDCN0001DPN |
| MC77 | OP869943 | NMDCN0001D18 | NMDCN0001D46 | NMDCN0001CUP | NMDCN0001CSF |
| MC78 | OP869944 | NMDCN0001D19 | NMDCN0001D47 | NMDCN0001CUQ | NMDCN0001CSG |
| MC79 | OP869945 | NMDCN0001D1A | NMDCN0001D48 | NMDCN0001CUR | NMDCN0001CSH |

**Supplementary Table S3.**

ANI values (%) and DDH relatedness (%) among strains BC49, MC63, MC62, BC56, MJ37, MC77 and type strains of related species

| Strains | BC49 | | MC63 | | MC62 | | BC56 | | MJ37 | | MC77 | |
| --- | --- | --- | --- | --- | --- | --- | --- | --- | --- | --- | --- | --- |
|  | ANI | DDH | ANI | DDH | ANI | DDH | ANI | DDH | ANI | DDH | ANI | DDH |
| BC49 | 100.0 | 100.00 |  |  |  |  |  |  |  |  |  |  |
| MC63 | 88.8 | 37.2 | 100.0 | 100.0 |  |  |  |  |  |  |  |  |
| MC62 | 90.0 | 40.2 | 88.5 | 36.7 | 100.0 | 100.0 |  |  |  |  |  |  |
| BC56 | 90.0 | 40.2 | 88.6 | 36.6 | 99.2 | 93.0 | 100.0 | 100.0 |  |  |  |  |
| MJ37 | 89.9 | 40.2 | 88.6 | 36.6 | 99.0 | 91.9 | 99.1 | 92.1 | 100.0 | 100.0 |  |  |
| MC77 | 88.4 | 36.2 | 93.8 | 54.4 | 88.1 | 35.60 | 88.3 | 35.60 | 88.1 | 43.3 | 100 | 100 |
| *R. croatiense* 13^T^ | 88.8 | 37.4 | **93.8** | **54.2** | 88.5 | 36.7 | 88.6 | 36.7 | 88.6 | 36.8 | **98.96** | **85.0** |
| *R. acidisoli* FH23 | **94.4** | **57.3** | 88.1 | 35.5 | 89.4 | 38.7 | 89.4 | 38.6 | 89.5 | 38.7 | 88.4 | 36.6 |
| *R. aegyptiacum* 950 | 86.7 | 32.6 | 87.6 | 34.2 | 87.1 | 33.4 | 87.1 | 33.5 | 87.1 | 33.6 | 88.2 | 35.8 |
| *R. aethiopicum* HBR26^T^ | 88.0 | 35.1 | 88.6 | 36.6 | 88.2 | 36.0 | 88.2 | 36.0 | 88.3 | 36.1 | 88.3 | 35.8 |
| *R. anhuiense* CCBAU 23252^T^ | 88.4 | 36.4 | 87.1 | 33.4 | 90.3 | 41.9 | 90.4 | 41.9 | 90.3 | 41.8 | 87.9 | 35.2 |
| *R. bangladeshense* BLR175^T^ | 86.6 | 32.2 | 87.5 | 34.0 | 86.8 | 32.6 | 86.8 | 32.7 | 86.8 | 32.7 | 88.0 | 35.0 |
| *R. binae* BLR195^T^ | 87.5 | 34.4 | 91.2 | 43.1 | 87.5 | 34.0 | 87.7 | 34.1 | 87.5 | 34.1 | 91.6 | 45.0 |
| *R. changzhiense* WYCCWR 11279^T^ | 88.5 | 36.6 | 87.0 | 33.4 | 90.1 | 40.9 | 90.1 | 41.0 | 90.2 | 41.0 | 88.0 | 35.4 |
| *R. chutanense* C5^T^ | 89.4 | 38.4 | 88.2 | 35.4 | **90.2** | **44.3** | **91.4** | **44.5** | **91.2** | **44.5** | 88.1 | 35.5 |
| *R. ecuadorense* CNPSO 671^T^ | 92.1 | 47.4 | 88.7 | 37.2 | 89.8 | 40.1 | 89.9 | 40.1 | 90.0 | 40.2 | 88.7 | 36.9 |
| *R. esperanzae* CNPSo 668^T^ | 87.1 | 33.8 | 88.8 | 37.0 | 87.4 | 34.1 | 87.5 | 34.3 | 87.4 | 34.4 | 88.8 | 37.1 |
| *R. etli* NBRC 15573^T^ | 87.3 | 33.5 | 89.1 | 37.7 | 87.3 | 33.8 | 87.3 | 33.7 | 87.3 | 33.7 | 89.3 | 38.0 |
| *R. hidalgonense* FH14^T^ | 90.5 | 41.5 | 88.0 | 35.3 | 90.0 | 40.4 | 89.9 | 40.4 | 90.0 | 40.4 | 87.9 | 35.0 |
| *R. indigoferae* CCBAU 71042^T^ | 88.4 | 36.7 | 86.9 | 33.5 | 89.7 | 40.5 | 89.8 | 40.5 | 89.7 | 40.5 | 87.7 | 35.1 |
| *R.* *laguerreae* CECT 8280^T^ | 88.1 | 36.0 | 86.7 | 33.0 | 89.3 | 39.2 | 89.3 | 39.1 | 89.4 | 39.2 | 87.7 | 34.9 |
| *R. leguminosarum* GLR17 | 88.4 | 36.8 | 87.2 | 34.1 | 89.5 | 39.8 | 89.6 | 40.1 | 89.6 | 40.0 | 88.0 | 35.5 |
| *R. lentis* BLR27^T^ | 87.0 | 33.2 | 87.7 | 34.6 | 87.6 | 34.2 | 87.6 | 34.3 | 87.6 | 34.3 | 88.3 | 35.8 |
| *R. phaseoli* NBRC 14785^T^ | 88.6 | 37.0 | 91.7 | 45.5 | 88.3 | 36.1 | 88.3 | 36.1 | 88.2 | 36.1 | 92.0 | 46.8 |
| *R. pisi* DSM 30132^T^ | 87.2 | 33.7 | 86.8 | 33.0 | 88.3 | 36.6 | 88.5 | 36.5 | 88.4 | 36.6 | 87.4 | 34.2 |
| *R. ruizarguesonis* UPM1133^T^ | 88.3 | 36.5 | 87.0 | 33.6 | 89.6 | 40.1 | 89.6 | 40.0 | 89.8 | 40.1 | 87.8 | 35.0 |
| *R. sophorae* CCBAU 03386^T^ | 88.3 | 36.2 | 86.9 | 33.3 | 89.5 | 39.9 | 89.7 | 40.1 | 89.8 | 40.1 | 87.9 | 35.1 |
| *R. sophoriradicis* CCBAU 03470^T^ | 88.9 | 37.5 | 92.7 | 48.7 | 88.6 | 36.8 | 88.5 | 36.9 | 88.6 | 36.9 | 92.99 | 51.0 |


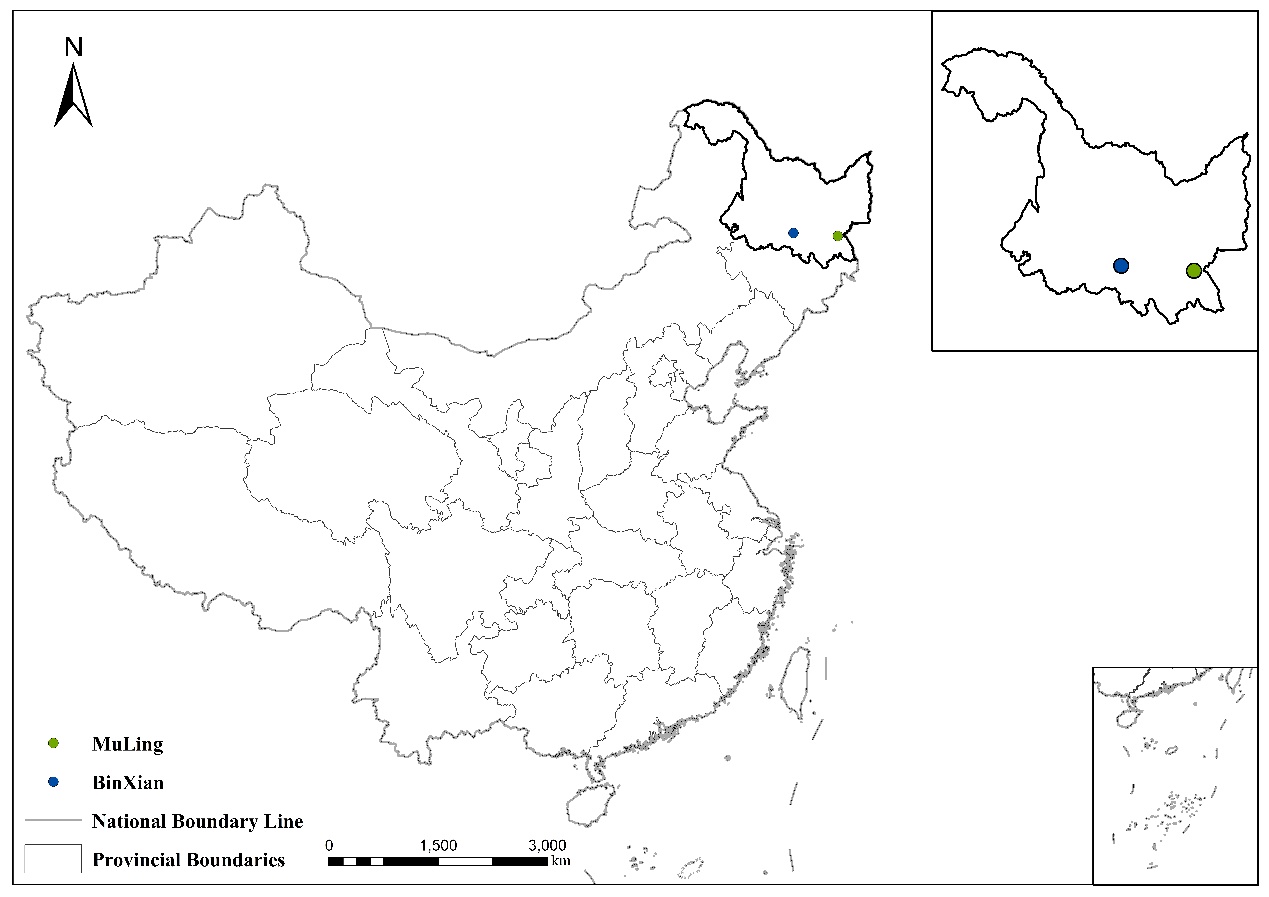


**Fig. S1.** The location of study area. The map is marked with the sampling place and the name of the sampling place


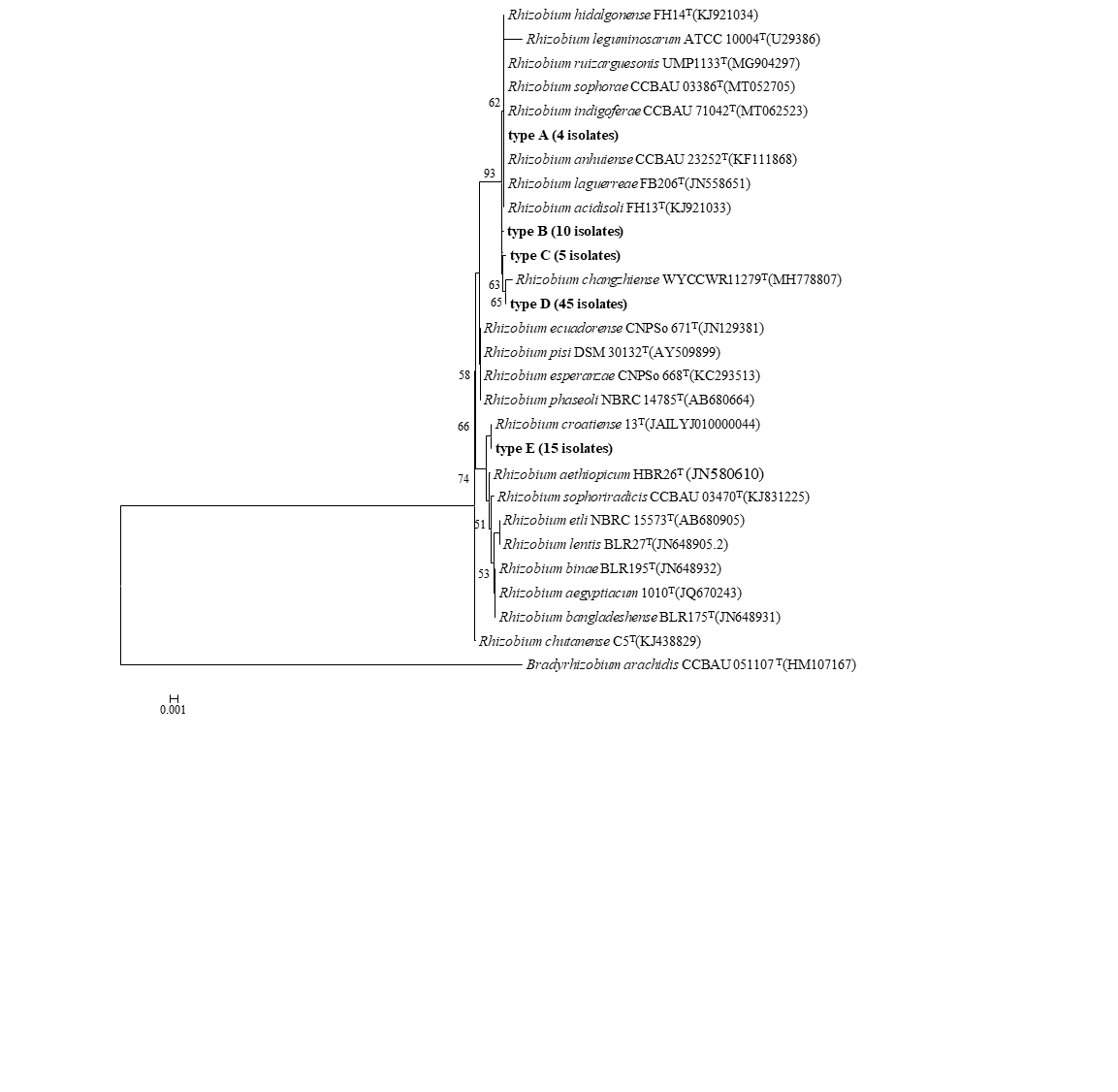


**Fig. S2.** Neighbor-joining tree showing the phylogenetic relationships of genotypes and phylogenetically related reference strains based upon the aligned 1175bp 16S rRNA gene sequences. Bar, 0.01% substitutions per site. *Bradyrhizobium* was taken as an outgroup. Higher than 50% bootstrap values are displayed.


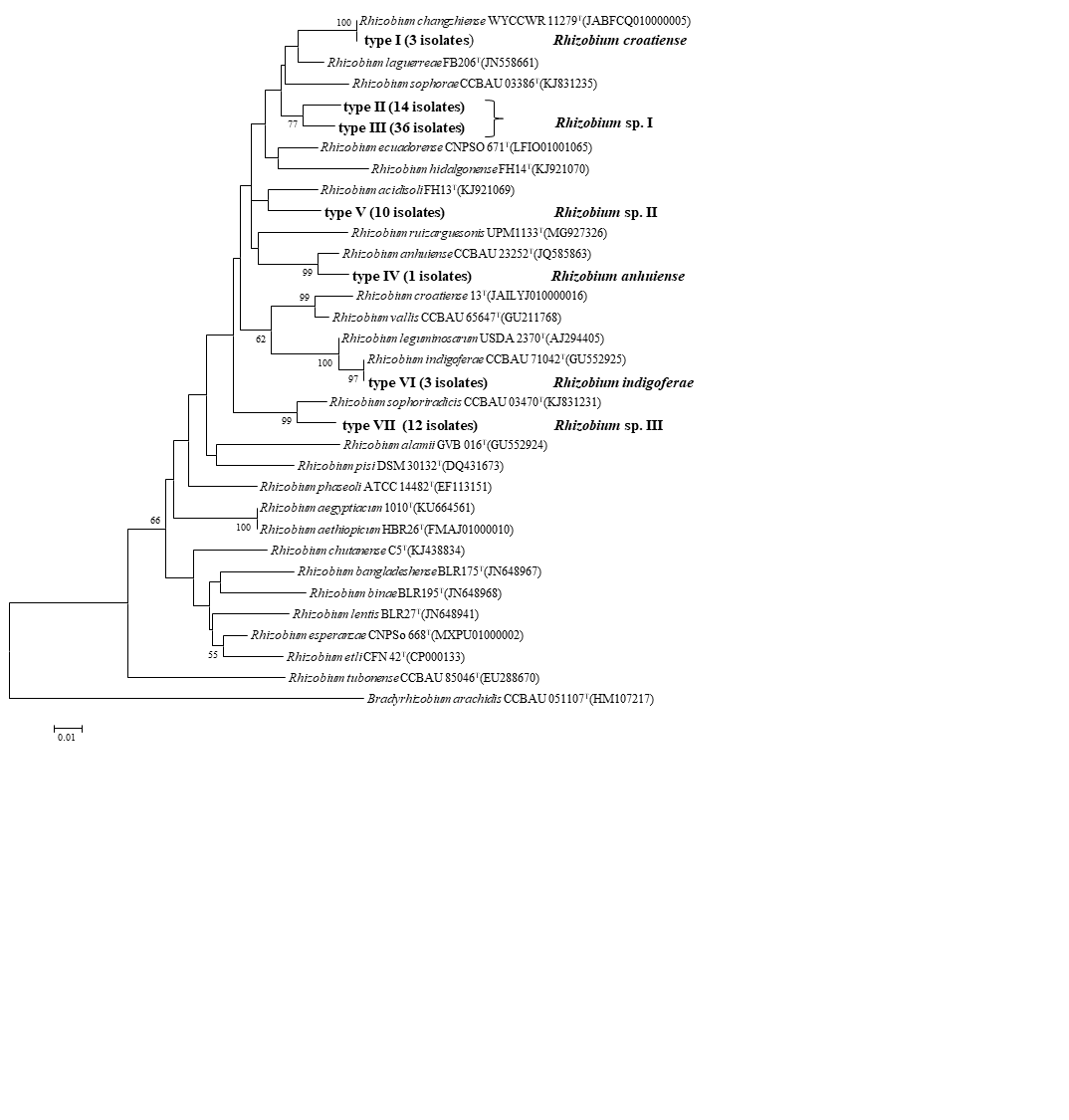


**Fig. S3.** Neighbor-joining tree showing the phylogenetic relationships of genotypes and phylogenetically related reference strains based upon the aligned 580bp concatenated sequences of *recA* and *atpD* genes. Bar, 1% substitutions per site. *Bradyrhizobium* was taken as an outgroup. Higher than 50% bootstrap values are displayed.


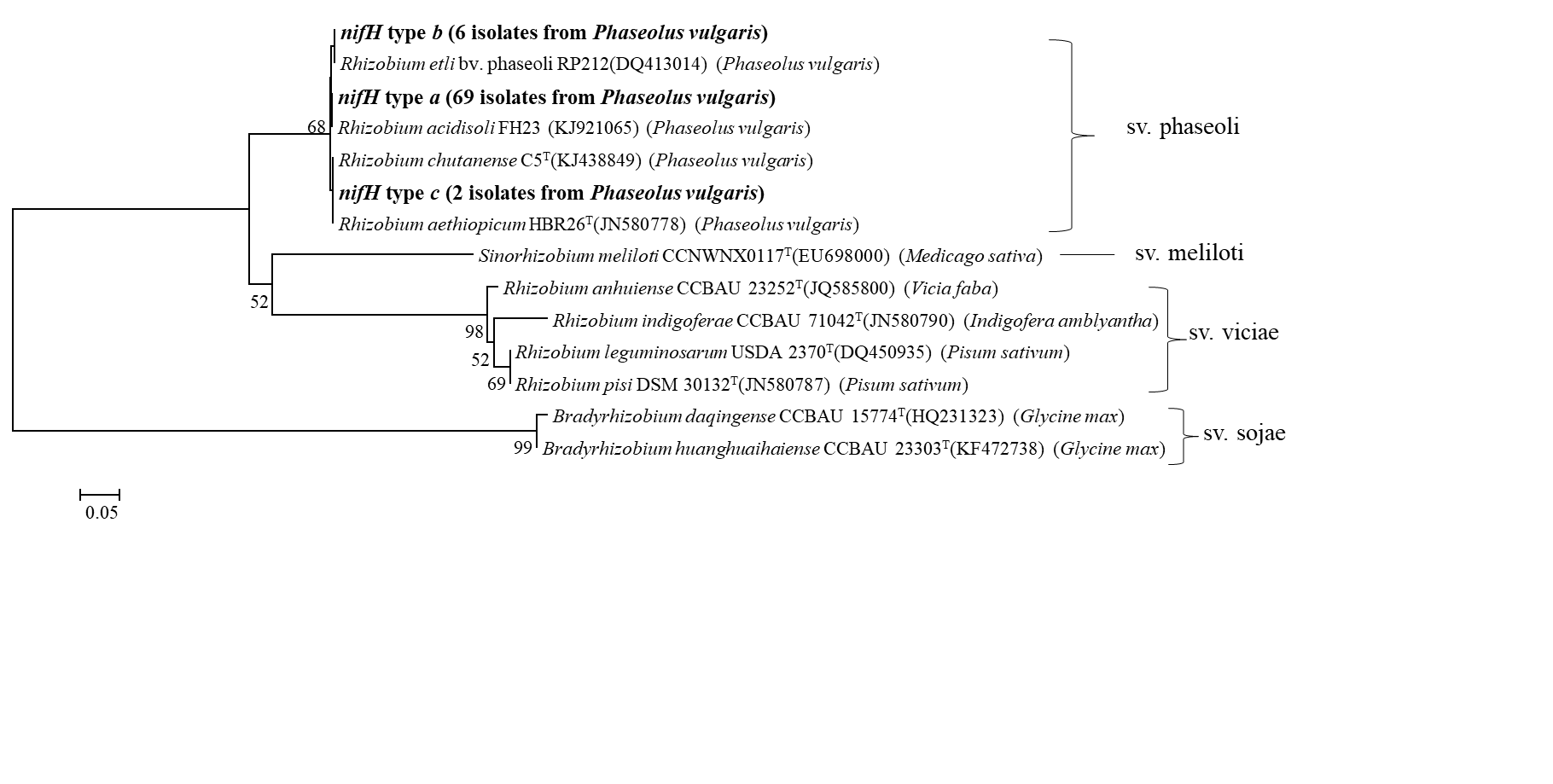


**Fig. S4.** Neighbor-joining tree showing the phylogenetic relationships of genotypes and phylogenetically rhizobia strains from nodules of variety legumes based upon the aligned 339bp *nifH* gene sequences. Bar, 5% substitutions per site. *Bradyrhizobium* was taken as an outgroup. Higher than 50% bootstrap values are displayed.
